# Effects of ChatGPT-generated immediate feedback integrated into VR-based OSCEs on nursing students’ performance: a randomized crossover study

**DOI:** 10.1186/s12912-026-04633-9

**Published:** 2026-04-13

**Authors:** Xinjia Dai, Yaodong Gong, Ling-Yun Ma

**Affiliations:** 1 Department of Neurology, The First Affiliated Hospital of Soochow University, No. 899, Pinghai Road, Suzhou, Jiangsu China; 2 Department of Pediatrics, Kunshan Women and Children’s Healthcare Hospital, No.5, Qingyang Road, Kunshan, Jiangsu China

**Keywords:** ChatGPT, Virtual reality, Objective structured clinical examination, Communication skills, Nursing education, Immediate feedback, Artificial intelligence-assisted learning

## Abstract

**Background:**

The Objective Structured Clinical Examination (OSCE) is a gold-standard assessment in nursing education, yet traditional and virtual reality (VR) formats often lack timely, individualized feedback, potentially limiting gains in higher-order competencies such as communication and decision-making. Advances in artificial intelligence (AI), particularly ChatGPT, offer opportunities to address this gap. To evaluate the impact of ChatGPT-generated immediate feedback integrated into VR-based OSCE stations on nursing students’ communication performance, clinical decision-making accuracy, and related learning outcomes.

**Methods:**

A randomized, two-period crossover trial was conducted among 65 final-year nursing students at a tertiary teaching hospital in China between March and May 2025. Participants completed two matched OSCE stations under VR-only and VR + ChatGPT conditions, with a one-week washout period between the two study phases. Primary outcomes included OSCE communication scores, clinical decision-making accuracy, and task completion time. Secondary outcomes comprised learning satisfaction, acceptance of AI-assisted feedback, academic and communication self-efficacy, reflection quality, and cognitive workload. Data were analyzed using paired statistical comparisons and linear mixed-effects models adjusting for sequence and period effects.

**Results:**

Compared with VR-only, VR + ChatGPT significantly improved communication scores (mean difference [MD] = 3.38, 95% CI: 2.12–4.64, *P* < 0.001, Cohen’s d = 0.63) and decision accuracy (MD = 3.91%, 95% CI: 2.09–5.72, *P* < 0.001, Cohen’s d = 0.49), while reducing completion time (MD = − 0.78 min, 95% CI: − 1.12 to − 0.44, *P* < 0.001, Cohen’s d = − 0.62). VR + ChatGPT also yielded higher learning satisfaction (MD = 0.31, *P* < 0.001), greater reflection quality (MD = 0.62, *P* < 0.001), lower cognitive workload (MD = − 5.7, *P* = 0.001), and significant gains in academic and communication self-efficacy (both *P* < 0.001). No severe adverse events occurred. Effects were consistent across subgroups defined by prior VR experience and digital literacy.

**Conclusions:**

Integrating ChatGPT-generated immediate feedback into VR OSCE scenarios significantly enhances nursing students’ communication and decision-making performance, accelerates skill acquisition, and reduces cognitive strain, without compromising safety.

**Clinical trial number:**

Not applicable.

**Supplementary Information:**

The online version contains supplementary material available at 10.1186/s12912-026-04633-9.

## Introduction

The Objective Structured Clinical Examination (OSCE) is widely recognized as a gold-standard method for assessing the clinical competencies of nursing students, encompassing essential domains such as communication, clinical reasoning, and procedural skills [1–3]. By employing standardized stations and structured scoring rubrics, OSCEs ensure objective and reproducible evaluations of both technical and non-technical abilities, which are critical for delivering safe and effective patient care [4–6]. Notably, OSCEs are particularly well suited for evaluating complex, integrative competencies—such as patient-centered communication and clinical decision-making—that require the application of knowledge, judgment, and interpersonal skills in real time.

In recent years, virtual reality (VR) simulation has emerged as a promising educational modality for OSCE implementation, offering immersive and interactive environments that replicate complex clinical scenarios without risk to patients [7–9]. Compared with traditional simulation approaches, VR-based training enables repeated practice, standardized scenario delivery, and controlled exposure to rare or high-acuity situations. These advantages have been associated with improvements in psychomotor skills, clinical decision-making, and learner engagement [10, 11]. However, despite these benefits, many VR scenarios rely on pre-programmed pathways and provide limited or delayed instructor feedback. Such constraints may reduce their effectiveness in fostering higher-order competencies, particularly patient-centered communication and adaptive diagnostic reasoning, which are highly dependent on timely, specific, and context-sensitive feedback.

Advances in artificial intelligence (AI), particularly large language models such as ChatGPT, have introduced new opportunities to address this limitation by enabling real-time, context-specific feedback during simulation-based learning [12]. By processing learner inputs and generating adaptive responses, AI-driven feedback can reinforce correct actions, identify areas for improvement, and promote reflective practice—features that are especially valuable for communication skills training, where nuanced and responsive feedback is essential [13, 14]. Despite these possibilities, research on integrating AI-based immediate feedback into VR simulation for nursing education remains scarce. Nevertheless, empirical evidence on the integration of AI-generated immediate feedback into VR-based OSCEs in nursing education remains limited.

Given substantial inter-individual variability in communication skills, clinical reasoning, and digital literacy among nursing students, a randomized crossover design was adopted. By allowing each participant to serve as their own control, this approach reduces confounding from stable individual differences and improves statistical efficiency, and has been widely used in simulation-based educational research with heterogeneous samples.

Previous studies in nursing and health professions education have typically employed brief VR-based simulation sessions (approximately 10–20 min) targeting specific clinical or communication skills [7–9]. Such short, immersive interventions are sufficient to produce measurable improvements in performance and engagement while limiting fatigue and cognitive load. In simulation-based crossover studies, washout periods of about one week are commonly used to reduce short-term carryover effects related to task familiarity and memory. This duration is generally considered adequate while remaining feasible within academic schedules; therefore, a one-week washout period was adopted in the present study.

Accordingly, the present study was designed to evaluate whether integrating ChatGPT-generated immediate feedback into VR-based OSCE stations improves nursing students’ communication performance, clinical decision-making accuracy, and related learning outcomes compared with VR simulation alone. Using a randomized crossover design, outcomes were compared between VR-only and VR + ChatGPT conditions across two matched OSCE stations. It was hypothesized that the VR + ChatGPT condition would be associated with higher OSCE communication scores and clinical decision-making accuracy, shorter task completion times, and greater learner satisfaction and acceptance of AI-assisted feedback. In addition, potential effect modification by baseline digital literacy and prior VR experience was explored.

## Methods

### Study design

A randomized, two-period crossover trial with an AB/BA sequence allocation was conducted to compare the effects of VR simulation combined with ChatGPT-generated immediate feedback (VR + ChatGPT) versus VR simulation alone (VR-only) on nursing students’ OSCE performance. Participants were randomly assigned in a 1:1 ratio to one of two sequences: Sequence AB (VR + ChatGPT in Period 1 followed by VR-only in Period 2) or Sequence BA (VR-only in Period 1 followed by VR + ChatGPT in Period 2). A one-week washout interval was implemented between the two study periods to minimize potential carryover effects and reduce learning or memory transfer between conditions. This crossover design allowed each participant to serve as their own control, thereby reducing between-subject variability and increasing statistical efficiency.

### Participants

Participants were recruited from the final-year Bachelor of Nursing program at *The First Affiliated Hospital of Soochow University* between March and May 2025. Eligible students were invited to participate via email announcements, classroom briefings, and postings on the university’s learning management system. Sample size estimation was based on detecting a mean paired difference of 3.0 points (SD = 6.0) in OSCE communication scores between VR + ChatGPT and VR-only conditions, derived from pilot data collected at *The First Affiliated Hospital of Soochow University* in 2024. With a two-tailed α of 0.05 and 80% power, the required sample size was calculated to be 58 participants. To account for an anticipated 10% attrition rate, a total of 65 participants were recruited.

### Sample size estimation

Sample size estimation was performed based on effect sizes reported in previous peer-reviewed studies evaluating communication performance in simulation-based and OSCE-related educational interventions. Prior research has commonly reported small-to-moderate within-participant improvements in OSCE communication scores when enhanced feedback or digital simulation tools were introduced, corresponding to standardized effect sizes of approximately 0.4–0.6 [15]. Assuming a mean paired difference of 3.0 points with a standard deviation of 6.0 (effect size ≈ 0.5), a two-sided significance level of 0.05, and 80% power, a minimum sample size of 54 participants was required for the crossover comparison. To account for potential attrition, the target sample size was increased to 65 participants.

### Inclusion criteria and exclusion criteria

Inclusion criteria were: (1) enrollment as a senior-year nursing student; (2) completion of all core clinical placement requirements; (3) basic computer literacy and ability to operate VR equipment; and (4) provision of written informed consent.

Exclusion criteria were: (1) self-reported vestibular disorders, severe visual impairment, or epilepsy that could interfere with VR use; (2) prior participation in an identical VR-based OSCE scenario within the past six months; and (3) unwillingness or inability to complete both crossover periods.

### Intervention conditions

Participants completed two standardized OSCE stations within a headset-based immersive virtual reality (VR) simulation environment under two experimental conditions: VR-only and VR + ChatGPT. The simulation was delivered through a head-mounted display (HMD), enabling fully immersive interaction with virtual patient avatars. The OSCE stations were designed to assess communication skills and clinical decision-making in common nursing scenarios (e.g., postoperative pain management and acute chest pain triage). Both stations were matched in complexity, duration, and scoring rubrics and were piloted prior to the study to ensure equivalence in difficulty and learning demands.

In the VR-only condition, participants engaged in a fully immersive VR simulation developed using Unity 3D software. Each scenario lasted approximately 10 min and involved pre-programmed patient avatars capable of scripted verbal and non-verbal interactions. Feedback in this condition was limited to automated scenario completion notifications (e.g., “Scenario complete”) and did not include performance-specific guidance related to communication or clinical reasoning.

In the VR + ChatGPT condition, participants interacted with the same VR scenarios supplemented with AI-generated immediate feedback. A ChatGPT model (OpenAI, GPT-4 architecture) was embedded into the VR platform via an application programming interface (API), enabling real-time processing of participants’ verbal and textual inputs. Although feedback was delivered through the avatar interface to preserve immersion, it was presented from a third-person, coaching-oriented perspective rather than as first-person patient dialogue. The avatar therefore functioned as a delivery interface rather than as the evaluative agent.

Feedback consisted of brief verbal prompts accompanied by concise on-screen text messages and was structured around predefined educational criteria, including communication clarity and empathy, clinical reasoning processes, patient safety considerations, and identification of missed or suboptimal decision points. Illustrative examples of feedback included statements such as: *“Consider clarifying your explanation to ensure patient understanding*,*” “You may wish to further assess symptom characteristics before determining priority*,*”* and *“Re-evaluate whether all potential safety concerns have been addressed.”* Importantly, feedback did not provide correct answers or prescribe specific actions but encouraged reflection and adaptive decision-making.

To minimize cognitive disruption, feedback was delivered during natural pauses in interaction and designed to be concise, supportive, and formative rather than directive. Task completion time was predefined as a primary study outcome and was automatically recorded by the VR system from scenario initiation to task completion. All participants received a standardized orientation session on VR equipment use and navigation prior to the first study session. A one-week washout period was implemented between experimental conditions to reduce potential carryover effects related to learning or memory. All OSCE sessions conducted under both conditions were designed exclusively for research and formative educational purposes. Performance scores and feedback generated during the study were not used for summative assessment, did not contribute to official course grades, and had no impact on participants’ academic progression.

No formal instructor-led debriefing sessions were conducted following the VR scenarios in either experimental condition. This design choice was intentional to isolate the effects of AI-generated immediate feedback and to avoid additional instructional influences that could confound outcome comparisons. Participants were allowed a brief rest period between sessions but did not receive structured reflective discussion or performance coaching from faculty during the study.

### Training protocol and feedback criteria

Under both experimental conditions, participants completed identical VR-based OSCE scenarios following a standardized training protocol. Prior to the first session, all participants received a brief orientation to VR equipment and scenario workflow. During each OSCE station, participants were required to complete core tasks including patient assessment, information gathering, clinical decision-making, and communication of care plans within a fixed time frame.

In the VR-only condition, participants progressed through scenarios without individualized instructional feedback, and interaction with the virtual patient followed pre-programmed scripts with only automated completion notifications provided.

In the VR + ChatGPT condition, AI-generated feedback was guided by predefined OSCE assessment domains focusing on communication quality, clinical reasoning processes, patient safety awareness, and task completeness. The system generated concise formative prompts during natural pauses based on participant inputs. Feedback highlighted strengths and areas for improvement while avoiding directive instructions or disclosure of correct responses, thereby maintaining the formative nature of the intervention.

### OSCE stations and scoring

Two parallel OSCE stations were developed to assess nursing students’ objective communication performance, clinical decision-making accuracy, and task completion time. Both stations were based on common clinical scenarios—postoperative pain assessment and acute chest pain triage—and were standardized in content, complexity, and duration. Detailed information regarding the validity and reliability of all measurement instruments is provided in Supplementary Material S1.

Communication performance was evaluated using a validated communication skills rating scale adapted from the Calgary–Cambridge Guide [1]. The scale comprised 10 items covering information gathering, empathy, clarity of explanations, and patient engagement, each rated on a 5-point Likert scale (1 = poor, 5 = excellent), yielding total scores ranging from 10 to 50. Two trained OSCE examiners, blinded to intervention allocation, independently rated each performance, with discrepancies resolved by consensus. Inter-rater reliability during the pilot phase was excellent (intraclass correlation coefficient = 0.89).

Clinical decision-making accuracy was defined as the proportion (%) of scenario-specific key decisions correctly completed, based on a checklist developed by a panel of three senior nurse educators. Each item was scored as correct (1) or incorrect (0), and accuracy was calculated as the percentage of correctly completed decisions. Task completion time was automatically recorded by the VR platform as the total elapsed time (seconds) from scenario initiation to completion of all required tasks, including patient interaction and decision-making.

OSCE performance scores were determined exclusively using standardized scoring rubrics applied by trained assessors blinded to experimental allocation. AI-generated feedback was not visible to assessors and was not incorporated into scoring criteria. Participants in the VR + ChatGPT condition were permitted to adjust their actions during the ongoing scenario in response to formative prompts; however, scores reflected observable performance outcomes rather than responsiveness to AI feedback. Accordingly, AI feedback functioned as formative instructional support rather than evaluative guidance.

### Cognitive workload

Cognitive workload was assessed using the NASA Task Load Index (NASA-TLX), a widely used subjective workload assessment tool developed by Hart and Staveland. The NASA-TLX evaluates perceived workload across six dimensions: mental demand, physical demand, temporal demand, performance, effort, and frustration. Participants completed the NASA-TLX immediately after each OSCE condition. A global workload score was calculated according to standard scoring procedures, with higher scores indicating greater perceived cognitive load. The NASA-TLX has demonstrated good validity and reliability in simulation-based and human–computer interaction research. In the present study, internal consistency reliability was satisfactory (Cronbach’s α = 0.86]).

### Secondary outcome measures

Learning satisfaction was assessed immediately after each OSCE station using a 5-item Likert scale developed for simulation-based education evaluation [1]. Items addressed perceived realism, engagement, perceived learning value, clarity of feedback, and overall satisfaction, each rated from 1 (“strongly disagree”) to 5 (“strongly agree”). The mean score across items was calculated for analysis, with higher values indicating greater satisfaction.

AI acceptance was measured only after the VR + ChatGPT condition using an adapted version of the Technology Acceptance Model (TAM) questionnaire [2]. The instrument included three subscales: perceived usefulness (PU, 4 items), perceived ease of use (PEOU, 4 items), and behavioral intention to use (BI, 3 items). All items were rated on a 7-point Likert scale (1 = “strongly disagree” to 7 = “strongly agree”), with subscale and total scores computed as the average of corresponding items.

Self-efficacy was evaluated pre- and post-intervention using the General Self-Efficacy Scale (GSES, 10 items) [3] and a scenario-specific Communication Self-Efficacy Scale (CSES, 6 items) developed for OSCE training contexts. Each item was rated from 1 (“not confident at all”) to 5 (“very confident”). Changes in self-efficacy scores before and after the study period were calculated to assess potential shifts in perceived competence.

### Adverse event monitoring

Virtual reality–induced motion sickness symptoms were systematically assessed immediately after each OSCE station using an 11-point Numerical Rating Scale (NRS; 0 = “no symptoms” to 10 = “worst possible symptoms”). Participants reporting NRS scores ≥ 7 were classified as having severe motion sickness. In addition, any other adverse events (e.g., dizziness, nausea, headache, eye strain) occurring during or after the intervention were documented by the study coordinator. Instances of intervention discontinuation, reasons for withdrawal, and time to symptom resolution were also recorded. All adverse events were reviewed by a faculty clinician to determine severity and relatedness to the intervention.

### Data anonymization and AI data handling

The integration of ChatGPT into the VR simulation platform was designed in compliance with institutional ethical approval and data protection requirements. No personally identifiable information (e.g., names, student identification numbers, or academic records) was collected or transmitted to the AI system. Prior to processing, all participant inputs were anonymized and limited to de-identified, task-specific information necessary for generating contextual feedback within the simulation. ChatGPT was accessed via an application programming interface (API), and AI-generated feedback was produced in real time without the storage of participant data on external servers beyond the duration of the interaction. Data transmitted to the AI system were not retained locally, linked to individual identities, or used for model training. All research data were stored securely on institutional servers in accordance with approved ethical protocols.

### Statistical analysis

All statistical analyses were performed using SPSS version 28.0 (IBM Corp., Armonk, NY, USA) and R version 4.3.2 (R Foundation for Statistical Computing, Vienna, Austria). Descriptive statistics were used to summarize participant characteristics and outcome measures. Continuous variables are presented as mean ± standard deviation (SD) or median with interquartile range (IQR), as appropriate, based on distribution normality assessed using the Shapiro–Wilk test. Categorical variables are reported as frequencies and percentages.

Given the randomized two-period crossover design, primary analyses focused on within-participant comparisons between the VR-only and VR + ChatGPT conditions. For primary outcomes—OSCE communication scores, clinical decision-making accuracy, and task completion time—paired t-tests were applied for normally distributed data, and Wilcoxon signed-rank tests were used when normality assumptions were not met.

To further account for crossover-specific effects, linear mixed-effects models (LMMs) were constructed for continuous outcomes, with participant identifier included as a random effect and intervention condition as the fixed effect of interest. Sequence (AB vs. BA) and period were included as covariates to control for potential sequence and period effects. For binary or proportional outcomes (e.g., decision accuracy), generalized linear mixed models (GLMMs) with a logit link function were applied using the same model structure.

Secondary outcomes, including learning satisfaction, acceptance of AI-assisted feedback, and changes in self-efficacy, were analyzed using the same analytical strategy, combining paired comparisons with mixed-effects modeling. Associations between primary and secondary outcomes were explored using Pearson or Spearman correlation coefficients, depending on data distribution.

Prespecified subgroup analyses were conducted to examine potential effect modification by prior VR experience (yes vs. no) and baseline digital literacy level (high vs. low). Interaction terms between intervention condition and subgroup variables were included in the mixed-effects models to test for differential intervention effects.

Potential carryover effects were assessed by comparing first-period outcomes between the two sequence groups. When evidence of carryover was detected (*P* < 0.05), sensitivity analyses restricted to first-period data were performed. Effect sizes are reported as mean differences with 95% confidence intervals (CI) for continuous outcomes, odds ratios (OR) for binary outcomes, and Cohen’s *d* where appropriate. All statistical tests were two-sided, and a P value < 0.05 was considered statistically significant.

## Results

### Participant recruitment and baseline characteristics

**Fig. 1:**
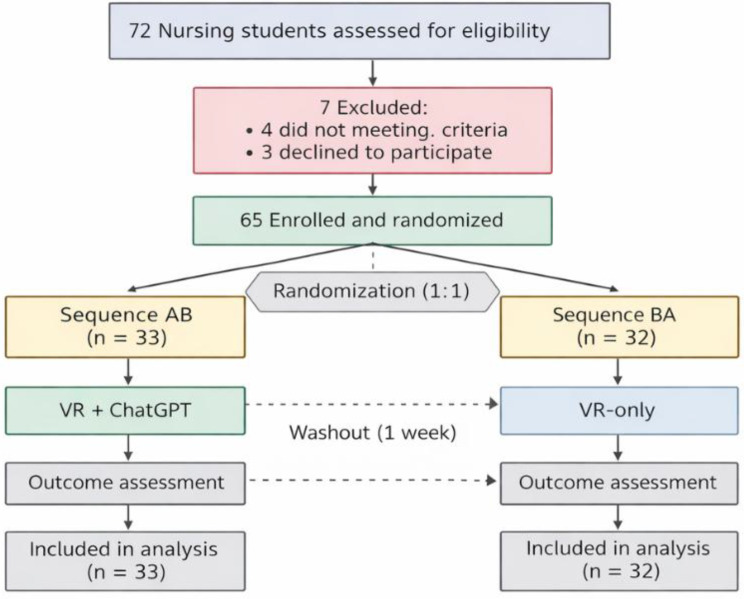
Study design and participant flow. A total of 72 nursing students were screened for eligibility, of whom 7 were excluded and 65 were enrolled. Participants were randomized to two sequences in a two-period crossover design comparing VR-only and VR+ChatGPT conditions: Sequence AB (VR+ChatGPT in Period 1 followed by VR-only in Period 2; n=33) and Sequence BA (VR-only in Period 1 followed by VR+ChatGPT in Period 2; n=32). A one-week washout period was applied between the two periods. Outcomes were assessed after each period, and all participants completed both periods and were included in the within-participant analysis

A total of 72 nursing students were screened for eligibility, of whom 65 met the inclusion criteria and were enrolled in the study (Fig. 1). All participants were randomized to one of two sequences: AB (VR + ChatGPT in Period 1, VR-only in Period 2; *n* = 33) or BA (VR-only in Period 1, VR + ChatGPT in Period 2; *n* = 32). All participants completed both OSCE stations and were included in the primary analysis, resulting in a data completion rate of 100%. No participant was lost to follow-up, and no data were missing for any primary or secondary outcomes. Baseline demographic and educational characteristics are presented in Table 1. The mean age of participants was 21.3 ± 1.1 years, and 84.6% were female. The average cumulative GPA was 3.45 ± 0.28 (on a 4.0 scale), and 43.1% reported prior experience with VR-based training. The median self-rated digital literacy score was 7 (IQR 6–8) on a 10-point scale, and 52.3% had previous OSCE experience. The distribution of prior VR experience, digital literacy, and OSCE experience did not differ significantly between the AB and BA sequences (all *P* > 0.05), indicating balanced baseline characteristics across groups.

### Primary outcomes

**Fig. 2:**
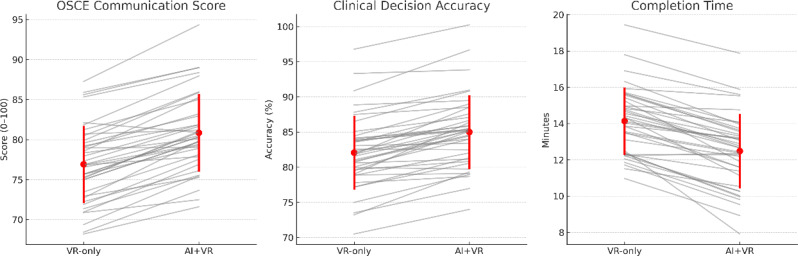
Paired scatter plots of primary outcomes. Paired scatter plots showing OSCE communication scores (**A**), clinical decision accuracy (**B**), and completion time (**C**) for the VR-only and VR + ChatGPT conditions. Each line represents an individual participant’s paired measurements, with black dots and error bars indicating the group mean ± 95% CI

Across the cohort, AI-augmented VR significantly outperformed VR-only on all three objective primary performance measures, including communication scores, clinical decision-making accuracy, and task completion time (Fig. 2; Table 2). For OSCE communication scores, the AI condition yielded higher scores (81.94 ± 6.13) than VR-only (78.56 ± 6.27), with a mean paired difference of 3.38 (95% CI = 2.12 to 4.64, *P* < 0.001; Cohen’s *d* = 0.63). In a mixed-effects model adjusting for period and sequence, the AI effect remained significant (β = 3.43, SE = 0.64, *P* < 0.001), with no evidence of period or sequence effects. For clinical decision accuracy, AI-augmented VR achieved higher accuracy (86.66 ± 7.35%) compared with VR-only (82.75 ± 7.63%), corresponding to a mean paired improvement of 3.91% points (95% CI = 2.09 to 5.72, *P* < 0.001; Cohen’s *d* = 0.49). This advantage persisted in a participant fixed-effects model with cluster-robust SEs (β = 3.68, SE = 0.92, *P* < 0.001). For completion time, participants completed tasks more quickly in the AI condition (9.89 ± 1.61 min) versus VR-only (10.67 ± 1.67 min), with a mean paired reduction of 0.78 min (95% CI = − 1.12 to − 0.44, *P* < 0.001; Cohen’s *d* = − 0.62). The time-saving effect was confirmed in the fixed-effects model (β = − 0.75, SE = 0.18, *P* < 0.001).

### Secondary outcomes

**Fig. 3:**
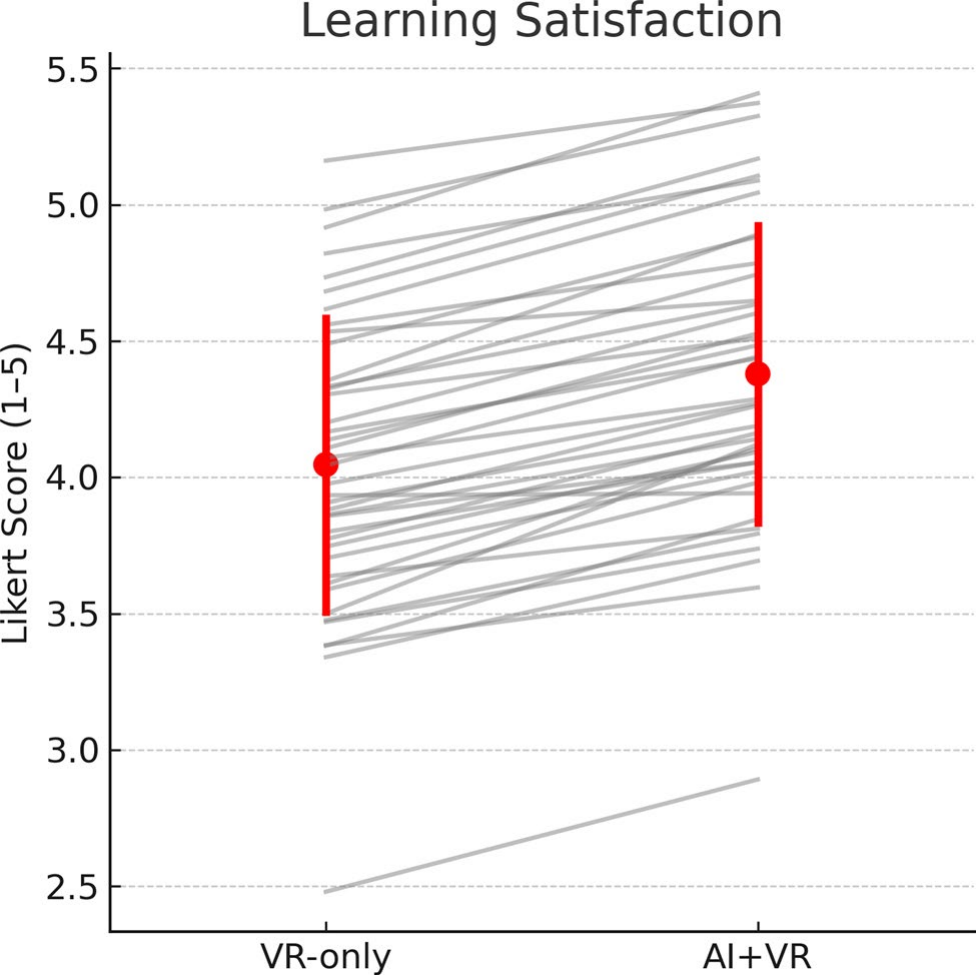
Paired comparison of learning satisfaction scores between conditions. Paired dot plot illustrating individual learning satisfaction scores under AI-augmented VR and VR-only conditions. Each line represents a participant’s score change across conditions. Mean scores (± 95% CI) are overlaid in red. Learning satisfaction was significantly higher in the AI-augmented VR condition (mean difference Δ = 0.31, 95% CI [0.21, 0.41], *P* < 0.001)

**Fig. 4:**
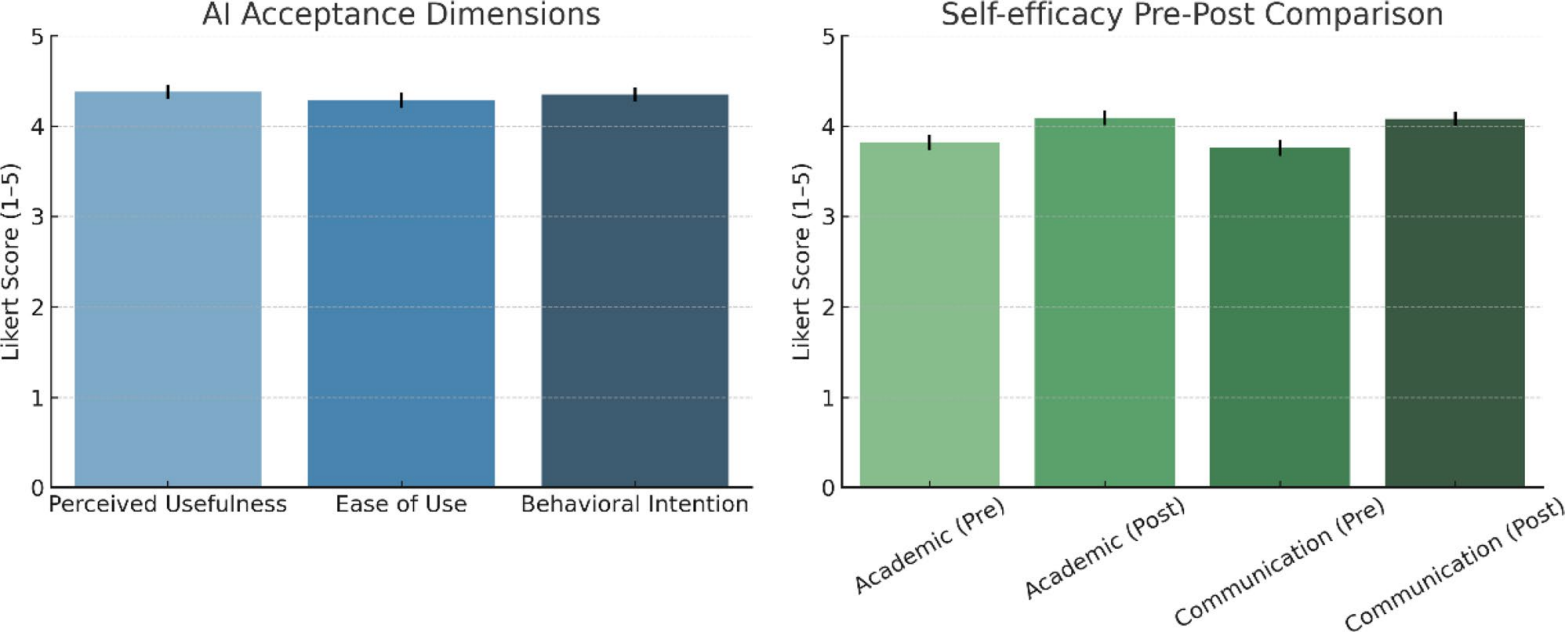
AI acceptance and self-efficacy outcomes. (left) Bar chart showing mean scores (± SD) for AI acceptance dimensions—perceived usefulness, perceived ease of use, and behavioral intention—across all participants. (right) Bar chart comparing pre- and post-intervention scores for academic and communication self-efficacy. Both self-efficacy domains showed significant improvement after the intervention (all *P* < 0.001)

Participants reported significantly higher learning satisfaction in the AI-augmented VR condition (4.42 ± 0.46) compared with VR-only (4.11 ± 0.52), with a mean paired increase of 0.31 points (95% CI = 0.21 to 0.41, *P* < 0.001; Cohen’s *d* = 0.65) (Table 3; Fig. 3). For AI acceptance, mean scores across the three Technology Acceptance Model (TAM) dimensions were: perceived usefulness = 4.38 ± 0.49, perceived ease of use = 4.29 ± 0.53, and behavioral intention = 4.35 ± 0.47. Subgroup analysis by baseline digital literacy (median split) indicated that participants with higher literacy reported greater perceived ease of use (mean difference = 0.24, 95% CI = 0.05 to 0.43, *P* = 0.014), whereas perceived usefulness and behavioral intention did not differ significantly between groups. Academic self-efficacy increased from 3.82 ± 0.54 to 4.09 ± 0.50 (Δ = 0.27, 95% CI = 0.18 to 0.36, *P* < 0.001), and communication self-efficacy rose from 3.76 ± 0.56 to 4.08 ± 0.51 (Δ = 0.32, 95% CI = 0.21 to 0.43, *P* < 0.001) (Fig. 4).

**Fig. 5:**
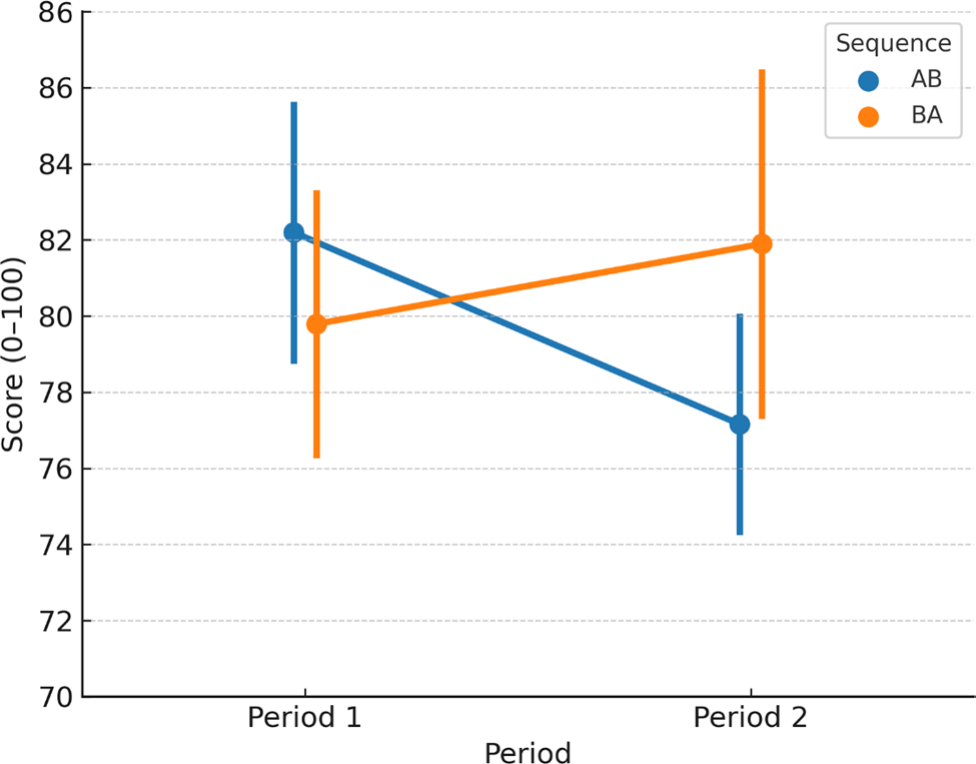
Sequence and period effects on OSCE communication scores in the crossover trial. Mean OSCE communication scores (± SD) are plotted by period for each sequence group: AB (VR + ChatGPT in Period 1, VR-only in Period 2) and BA (VR-only in Period 1, VR + ChatGPT in Period 2). Both sequences demonstrated higher scores in the VR + ChatGPT condition regardless of order, with no significant sequence or carryover effects detected in the linear mixed-effects models (*P* > 0.05 for all)

In the linear mixed-effects models for each primary outcome, the sequence effect (order of exposure: AB vs. BA) was not statistically significant for OSCE communication scores (β = 0.42, SE = 0.58, *P* = 0.48), clinical decision accuracy (β = 0.63, SE = 0.72, *P* = 0.39), or completion time (β = − 0.18, SE = 0.31, *P* = 0.57)(Fig. 5).

### Correlation and exploratory analyses

**Fig. 6:**
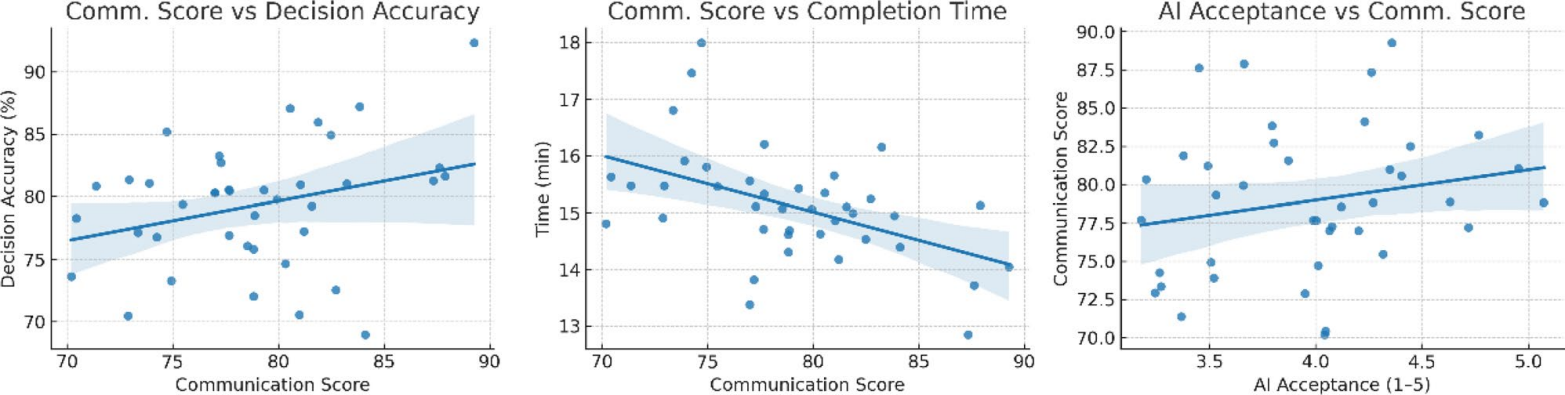
Correlation scatter plots between primary and secondary outcomes. Pearson correlation plots showing the relationships between OSCE communication scores, clinical decision accuracy, completion time, and AI acceptance scores. Communication scores were positively associated with decision accuracy (*r* = 0.46, *P* < 0.001) and AI acceptance (*r* = 0.41, *P* < 0.001), and negatively associated with completion time (*r* = − 0.28, *P* = 0.017). Decision accuracy was also inversely related to completion time (*r* = − 0.31, *P* = 0.009)

**Fig. 7:**
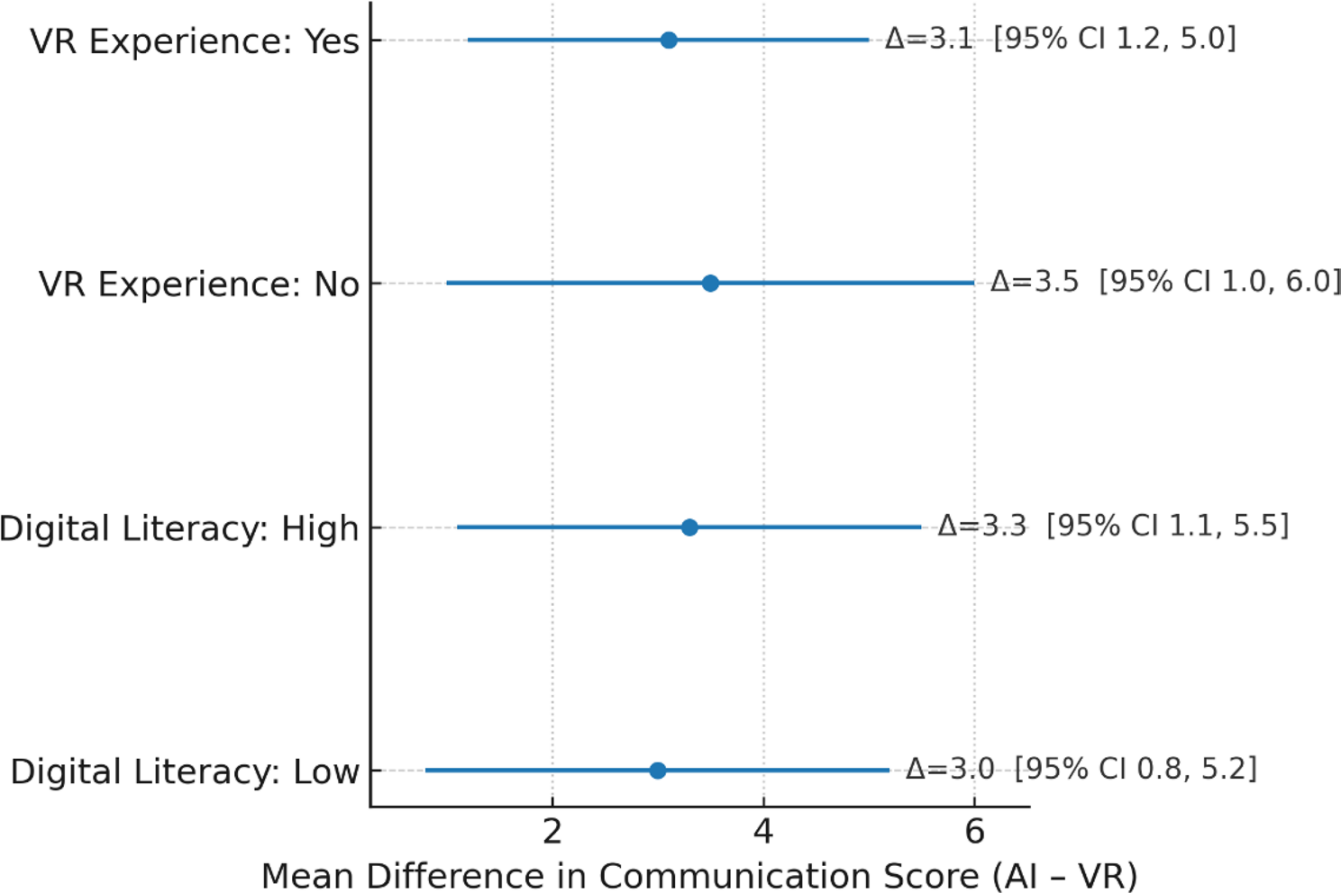
Subgroup forest plot of intervention effects on OSCE communication scores. Forest plot showing mean differences (VR + ChatGPT – VR-only) in OSCE communication scores with 95% confidence intervals across predefined subgroups. Subgroup categories include VR experience (yes/no) and digital literacy (high/low). Intervention effects were consistent across all subgroups, with no significant interaction effects detected (*P* > 0.05 for all)

**Fig. 8:**
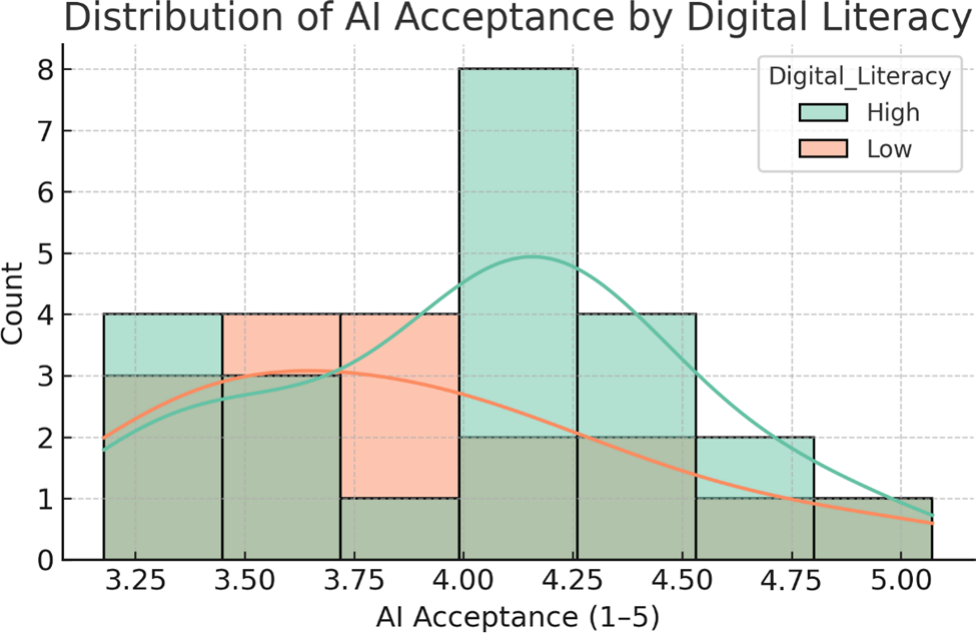
Distribution of AI acceptance scores by digital literacy level. Histogram comparing AI acceptance scores between high and low digital literacy groups. Although the high digital literacy group showed a slightly higher mean acceptance score, the difference was not statistically significant (*P* = 0.21)

Pearson correlation analyses demonstrated a moderate positive association between OSCE communication scores and clinical decision accuracy (*r* = 0.46, *P* < 0.001), alongside a weak negative association between communication scores and completion time (*r* = − 0.28, *P* = 0.017). Clinical decision accuracy was also inversely correlated with completion time (*r* = − 0.31, *P* = 0.009). AI acceptance (overall mean score) correlated positively with both communication scores (*r* = 0.41, *P* < 0.001) and decision accuracy (*r* = 0.38, *P* = 0.002), but showed no significant association with completion time (*r* = − 0.15, *P* = 0.21) (Fig. 6). Subgroup analyses indicated that the beneficial effect of VR + ChatGPT over VR-only on communication scores was consistent across participants with and without prior VR experience (interaction *P* = 0.62) and across high vs. low digital literacy groups (interaction *P* = 0.57). Similar consistency was observed for clinical decision accuracy and completion time, suggesting that neither prior VR exposure nor digital literacy significantly moderated the intervention effect (Fig. 7). A further exploratory comparison of AI acceptance scores by digital literacy level showed a slightly higher mean in the high-literacy group; however, the difference did not reach statistical significance (*P* = 0.21) (Fig. 8).

### Adverse events and withdrawal rates

**Fig. 9:**
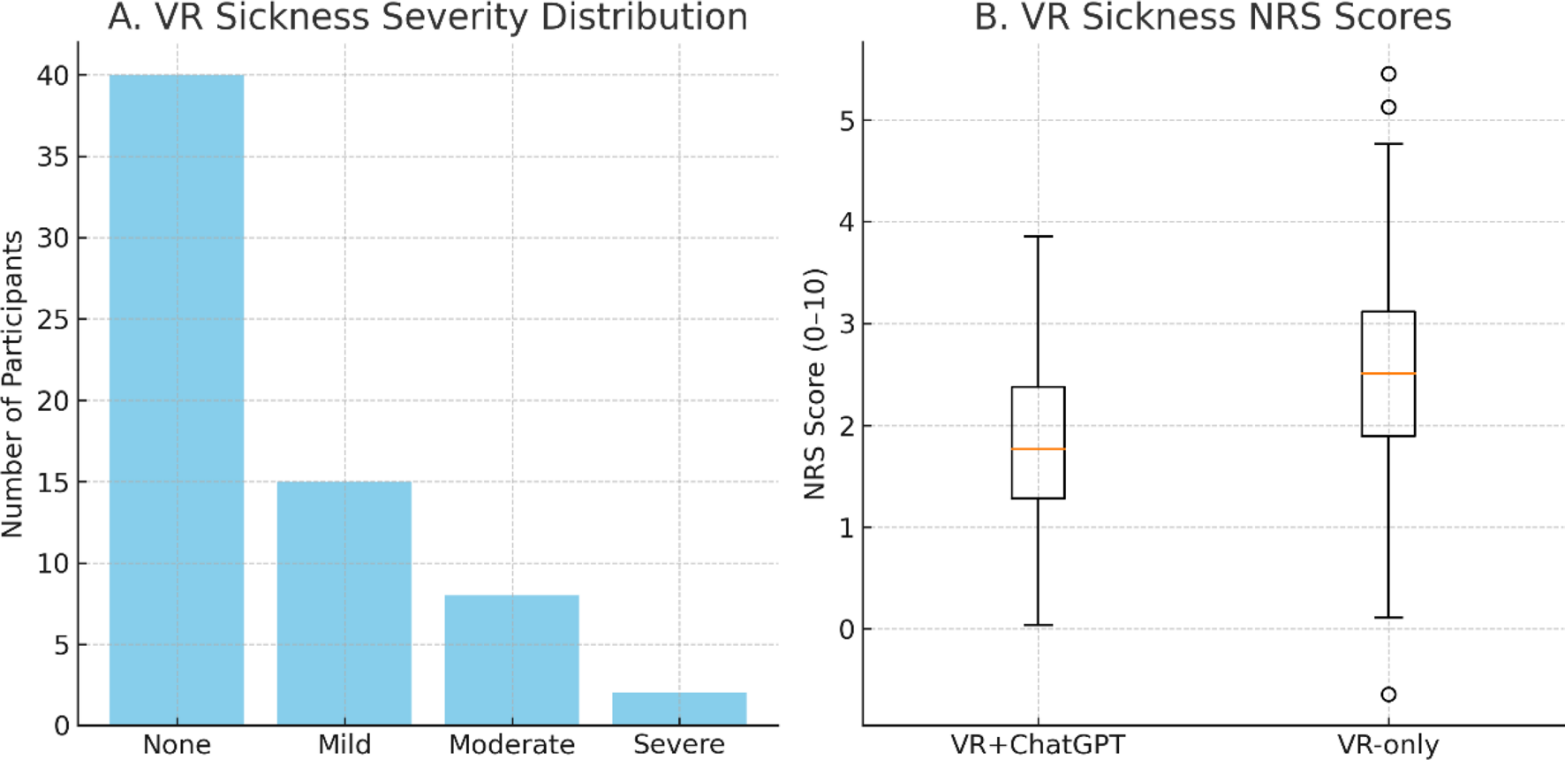
Adverse events and VR sickness severity. (**A**) Distribution of VR-induced motion sickness severity levels across all participants, categorized as none, mild, moderate, or severe. (**B**) Boxplot of numeric rating scale (NRS, 0–10) scores for VR-induced motion sickness in the VR + ChatGPT and VR-only conditions. A total of six participants (9.2%) reported mild-to-moderate symptoms (median NRS score = 3, IQR = 2–4), with no cases of severe motion sickness (NRS ≥ 7). All symptoms resolved spontaneously within five minutes post-task. Intervention discontinuation occurred in two participants (3.1%), both in the VR-only condition; both resumed after rest and completed the second session. No serious adverse events were observed

Adverse events and withdrawal data are presented in Table 4 and visualized in Fig. 9. Six participants (9.2%) reported mild-to-moderate VR-induced motion sickness symptoms, with a median NRS score of 3 (IQR: 2–4). All cases resolved spontaneously within five minutes post-task, without requiring medical intervention. No severe motion sickness (NRS ≥ 7) or serious adverse events occurred during the study. Intervention discontinuation due to discomfort was reported in two participants (3.1%), both in the VR-only condition; both resumed participation after a brief rest and successfully completed their subsequent session. The incidence of motion sickness was similar between conditions (VR + ChatGPT: 4.6% vs. VR-only: 6.2%).

### Learning curve, reflection quality, and cognitive load

**Fig. 10:**
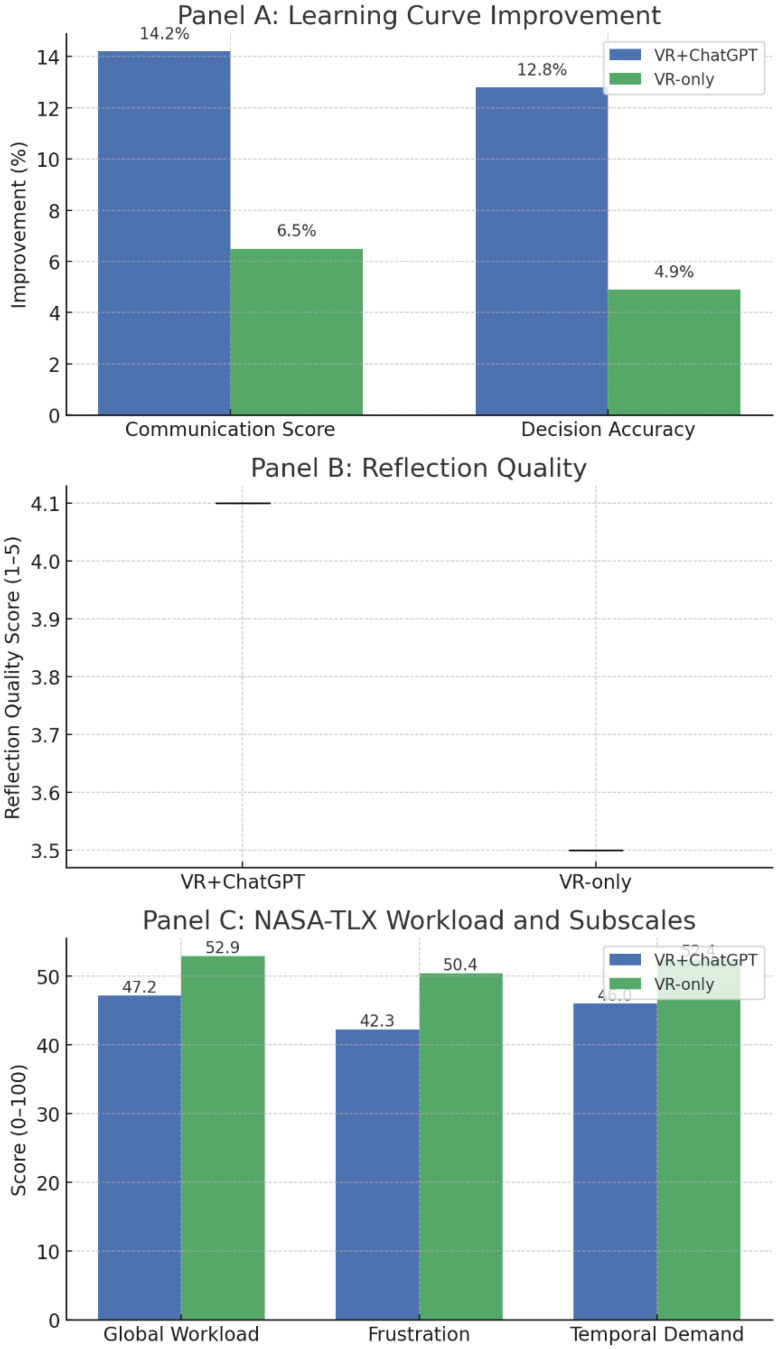
Extended analyses: learning curve, reflection quality, and cognitive load. Panel A: Mean improvement (%) in OSCE communication scores and clinical decision accuracy from first to second encounter in VR + ChatGPT and VR-only conditions. Panel B: Boxplots of reflection quality scores between conditions. Panel C: Bar plots of NASA-TLX global workload scores and key subscales (frustration, temporal demand)

Participants in the VR + ChatGPT condition demonstrated a steeper performance improvement across both OSCE stations compared with VR-only, with mean communication scores increasing by 14.2% (95% CI: 10.1–18.3%) versus 6.5% (95% CI: 3.0–10.0%) and clinical decision accuracy improving by 12.8% versus 4.9% (interaction *P* = 0.012 and *P* = 0.021, respectively). Reflection narratives scored via a 5-point structured rubric were significantly higher in the AI-assisted condition (4.1 ± 0.6 vs. 3.5 ± 0.7; Δ = 0.62, 95% CI: 0.48–0.76, *P* < 0.001), with the greatest gain in the “analysis and synthesis” domain. Cognitive workload measured by NASA-TLX was lower in the VR + ChatGPT group (47.2 ± 12.5 vs. 52.9 ± 13.1; Δ = − 5.7, 95% CI: − 8.9 to − 2.5, *P* = 0.001), driven mainly by reductions in “frustration” (–8.1 points, *P* < 0.001) and “temporal demand” (–6.4 points, *P* = 0.004)(Table 5; Fig. 10).

## Discussion

This crossover study demonstrated that the integration of ChatGPT-based immediate feedback into VR simulation significantly enhanced nursing students’ objective OSCE performance, particularly in communication and clinical decision-making domains assessed using standardized scoring rubrics. Compared with VR simulation alone, the VR + ChatGPT condition yielded higher communication scores and greater decision accuracy, while also reducing task completion time. These improvements were consistent across subgroups defined by prior VR experience and digital literacy levels, with no significant sequence or carryover effects observed. Additionally, participants reported high learning satisfaction and generally positive attitudes toward AI-assisted training, indicating both educational efficacy and user acceptance of the intervention.

The improvement was observed in objective performance metrics rather than solely in self-reported perceptions, strengthening the validity of the findings. The observed performance gains in the VR + ChatGPT condition may be explained by the educational benefits of immediate, individualized feedback [16, 17]. ChatGPT’s natural language processing capabilities enable timely, context-specific guidance during the OSCE scenario, allowing learners to identify communication gaps, clarify clinical reasoning, and promptly adjust their strategies [18]. This aligns with experiential learning theory, where iterative reflection and active problem-solving consolidate knowledge and skill acquisition. Moreover, AI-generated feedback reduces cognitive load by structuring information into actionable points, potentially accelerating the transition from novice to competent performance [19, 20]. The immersive VR environment may further amplify these effects by providing realistic, consequence-driven practice opportunities that reinforce learning through repetition and contextual relevance [21, 22].

The current results reinforce and extend prior evidence on VR simulation and AI-enhanced learning in health professions education. Previous randomized and quasi-experimental studies have demonstrated that VR-based OSCE training improves psychomotor performance, procedural accuracy, and clinical decision-making in nursing and medical students; however, the effects on communication skills have been more variable. One explanation lies in the absence of real-time, context-specific feedback—VR alone often provides immersive practice but leaves learners reliant on delayed debriefings, which may reduce the immediacy of reflective learning [23–25]. Traditional OSCEs with faculty-led feedback, on the other hand, have shown consistent improvements in communication competence, but such approaches require significant human resources, are susceptible to inter-assessor variability, and may be impractical in large-scale training settings [26]. Our study differs from earlier VR-only or traditional OSCE research in two important ways. First, ChatGPT’s integration provided instantaneous, tailored feedback during the scenario rather than post-hoc, allowing for in-situ adjustment of communication strategies and decision pathways. This likely explains the larger effect sizes observed in our study compared to prior VR-only interventions. Second, the AI-driven feedback ensured standardization across learners, reducing variability in feedback quality that is often present in human-led sessions. These combined advantages may explain why the VR + ChatGPT condition produced consistent benefits across both novice and more digitally literate students, with no significant interaction effects by prior VR exposure—an observation not commonly reported in prior simulation literature.

The educational significance of these findings is multifaceted. First, the observed improvement in OSCE communication scores and decision accuracy under the VR + ChatGPT condition supports the integration of AI-assisted feedback into competency-based nursing curricula [27]. Such integration addresses a critical bottleneck in OSCE-based training: the limited availability of qualified assessors to provide immediate, individualized guidance. Second, the intervention’s efficiency benefits—evidenced by shorter completion times—suggest that institutions could train more students within the same OSCE schedule without sacrificing performance quality [28]. From a pedagogical standpoint, the capacity for ChatGPT to adapt feedback to the learner’s level and errors supports a personalized learning paradigm, aligning with self-regulated learning theory [29]. Students receive direct, actionable cues that guide iterative improvement, potentially accelerating their trajectory from novice to proficient communicator and decision-maker. In addition, the high satisfaction and AI acceptance ratings observed in our study indicate that students perceive such hybrid training as both useful and engaging, which is critical for long-term adoption [30]. On a broader scale, the scalability of VR + ChatGPT training could have implications for resource-limited educational settings. By reducing reliance on faculty availability, this model can democratize access to high-fidelity simulation, particularly in remote or underserved regions [13]. Furthermore, the technology’s modularity allows for adaptation to various clinical disciplines and cultural contexts, making it a versatile tool in global health professions education. Finally, the standardized nature of AI-driven feedback offers potential advantages in assessment validity and inter-rater reliability—two persistent challenges in OSCE-based evaluation [31].

An apparent discrepancy between our findings and traditional cognitive load theory warrants consideration. Classical frameworks suggest that additional instructional input in immersive VR environments may increase cognitive workload, particularly intrinsic load related to complex clinical reasoning. However, we observed reduced perceived workload in the VR + ChatGPT condition. One possible explanation is the distinction between intrinsic and extraneous cognitive load. While task complexity imposed intrinsic demands, AI-generated feedback may have reduced extraneous load by structuring information, guiding attention, and reducing uncertainty during decision-making. Because feedback was delivered during natural interaction pauses rather than active task execution, cognitive disruption was minimized, allowing learners to integrate guidance efficiently. Thus, AI-assisted feedback may function as cognitive scaffolding rather than an additional burden, suggesting that the timing and format of feedback play an important role in regulating cognitive load in immersive simulation environments.

The reduction in task completion time under the VR + ChatGPT condition merits further consideration. Although additional feedback might intuitively be expected to prolong task execution, AI-generated prompts may have enhanced decisional efficiency by reducing uncertainty, structuring information processing, and guiding attention toward clinically relevant cues. By providing timely scaffolding, the system may have shortened periods of hesitation or unproductive exploration, thereby accelerating task progression. However, increased speed does not necessarily equate to deeper clinical reasoning. It remains possible that AI-assisted guidance may influence exploratory behavior or reduce divergent thinking, effects that are not fully captured by standardized OSCE scoring metrics. Additionally, subtle safety-related processes—such as identification of latent safety threats—may require more nuanced assessment tools beyond performance scores and completion time. Future research should therefore evaluate whether efficiency gains are accompanied by sustained reasoning depth and safety awareness in more complex clinical contexts.

Several limitations should be acknowledged. First, this single-site study was conducted within a research-oriented OSCE framework rather than a curriculum-embedded or summative setting, which may limit generalizability to routine practice. Second, although assessors were blinded, participants were aware of AI-assisted feedback, introducing potential performance or expectancy effects. Third, outcomes were measured immediately post-intervention, and long-term retention or transfer to clinical practice remains uncertain. In addition, AI-generated feedback was not formally benchmarked against expert feedback, and VR scenarios cannot fully replicate the complexity of real patient encounters. Future research should evaluate AI-assisted feedback within curriculum-integrated and real-world clinical training contexts using multi-site designs. Longitudinal studies are needed to assess retention and transfer, and benchmarking AI-generated feedback against expert standards will be essential, particularly for high-stakes applications. Qualitative investigations may further inform responsible integration of AI-assisted feedback into health professions education.

## Conclusion

In our study, integrating ChatGPT-generated real-time feedback into VR-based OSCE scenarios significantly improved nursing students’ communication scores and clinical decision-making accuracy, and modestly reduced task completion time, without increasing adverse events. The combined approach enhanced both objective performance and subjective learning experiences, indicating its potential as a scalable and individualized training method in nursing education. While findings are promising, broader multi-site validation, long-term outcome assessment, and rigorous evaluation of AI feedback quality are needed before widespread adoption. This study contributes to the growing evidence supporting AI-enhanced simulation as a complement to traditional clinical skills training, with potential implications for improving competency-based assessment and personalized learning in health professions education.

**Table 1:** Baseline characteristics of participants by sequence group

Variable	Total (*N* = 64)	AB sequence (*n* = 32)	BA sequence (*n* = 32)	*P* value
Age, years	21.43 ± 1.16	21.51 ± 1.17	21.34 ± 1.16	0.520
Gender: Female	56 (87.5%)	28 (87.5%)	28 (87.5%)	1.000
Gender: Male	8 (12.5%)	4 (12.5%)	4 (12.5%)	1.000
GPA	3.42 ± 0.27	3.44 ± 0.27	3.40 ± 0.28	0.480
Year in program: 3rd year	35 (54.7%)	18 (56.3%)	17 (53.1%)	0.808
Year in program: 4th year	29 (45.3%)	14 (43.8%)	15 (46.9%)	0.808
Clinical hours completed	421.14 ± 82.22	418.75 ± 80.54	423.53 ± 84.90	0.832
Prior VR experience: Yes	18 (28.1%)	9 (28.1%)	9 (28.1%)	1.000
Prior VR experience: No	46 (71.9%)	23 (71.9%)	23 (71.9%)	1.000
Prior online simulations	2.17 ± 1.50	2.25 ± 1.55	2.09 ± 1.47	0.692
Computer use, days/week	6.09 ± 1.08	6.06 ± 1.06	6.13 ± 1.12	0.766
VR use, times/month	1.00 ± 0.97	1.00 ± 1.00	1.00 ± 0.95	1.000
Digital literacy (0–10)	7.39 ± 1.11	7.47 ± 1.13	7.31 ± 1.10	0.532
Baseline self-efficacy (1–5)	3.36 ± 1.05	3.31 ± 1.05	3.41 ± 1.06	0.664
Baseline test anxiety score	42.28 ± 7.87	42.44 ± 8.15	42.13 ± 7.71	0.865
Previous OSCE experience: Yes	40 (62.5%)	21 (65.6%)	19 (59.4%)	0.610
Previous OSCE experience: No	24 (37.5%)	11 (34.4%)	13 (40.6%)	0.610
Specialty interest: Emergency	15 (23.4%)	7 (21.9%)	8 (25.0%)	0.978
Specialty interest: Med-Surg	17 (26.6%)	9 (28.1%)	8 (25.0%)	0.978
Specialty interest: Pediatrics	11 (17.2%)	6 (18.8%)	5 (15.6%)	0.978
Specialty interest: Community	11 (17.2%)	5 (15.6%)	6 (18.8%)	0.978
Specialty interest: ICU	9 (14.1%)	5 (15.6%)	4 (12.5%)	0.978

*P* values were calculated using independent-samples *t* test for continuous variables and χ² test (or Fisher’s exact test when appropriate) for categorical variable**s**

**Table 2:** Primary outcomes by condition, paired comparisons, and model-adjusted effects

Outcome	AI mean ± SD	VR mean ± SD	Paired mean difference (AI – VR)	95% CI	Paired *P* value	Cohen’s d	Model effect (AI vs. VR)	Model SE	Model *P* value
OSCE communication score (0–100)	81.94 ± 6.13	78.56 ± 6.27	3.38	[2.12, 4.64]	< 0.0001	0.63	3.43 (LMM)	0.64	< 0.0001
Clinical decision accuracy (%)	86.66 ± 7.35	82.75 ± 7.63	3.91	[2.09, 5.72]	< 0.0001	0.49	3.68 (OLS, FE)	0.92	< 0.0001
Completion time (minutes)	9.89 ± 1.61	10.67 ± 1.67	–0.78	[–1.12, − 0.44]	< 0.0001	–0.62	–0.75 (OLS, FE)	0.18	< 0.0001

Paired comparisons use paired t-tests with 95% confidence intervals. Cohen’s d denotes standardized effect size for within-subject designs

LMM = linear mixed-effects model with participant as a random intercept and fixed effects for condition (AI vs VR), period, and sequence

OLS, FE = ordinary least squares with participant fixed effects (categorical ID) and cluster-robust standard errors (clustered by participant), adjusting for period and sequence

Negative values for completion time indicate faster performance under the AI condition

**Table 3:** Secondary outcomes (means ± SD) and paired comparisons

Outcome	Condition / Timepoint	Mean ± SD	Mean difference (95% CI)	*P* value	Cohen’s d
Learning satisfaction (1–5)	**AI-augmented VR**	4.42 ± 0.46	0.31 [0.21, 0.41]	< 0.001	0.65
	**VR-only**	4.11 ± 0.52	—	—	—
Perceived usefulness (1–5)	**AI-augmented VR**	4.38 ± 0.49	—	—	—
Perceived ease of use (1–5)	**AI-augmented VR**	4.29 ± 0.53	High vs. low literacy: 0.24 [0.05, 0.43]	0.014	—
Behavioral intention (1–5)	**AI-augmented VR**	4.35 ± 0.47	—	—	—
Academic self-efficacy (1–5)	**Pre**	3.82 ± 0.54	0.27 [0.18, 0.36]	< 0.001	0.50
	**Post**	4.09 ± 0.50	—	—	—
Communication self-efficacy (1–5)	**Pre**	3.76 ± 0.56	0.32 [0.21, 0.43]	< 0.001	0.57
	**Post**	4.08 ± 0.51	—	—	—

*.*

**Table 4:** Adverse Events and Withdrawals

Adverse event	VR + ChatGPT (*n* = 65)	VR-only (*n* = 65)	Total (*n* = 65)
Any motion sickness, n (%)	3 (4.6%)	4 (6.2%)	6 (9.2%)
Median NRS score for motion sickness	3 (2–4)	3 (2–4)	3 (2–4)
Severe motion sickness (NRS ≥ 7), n	0	0	0
Intervention discontinued, n (%)	0	2 (3.1%)	2 (3.1%)
Serious adverse events, n	0	0	0

**Table 5:** Additional analyses of learning curve, reflection quality, and cognitive load

Outcome	VR + ChatGPT (Mean ± SD)	VR-only (Mean ± SD)	Mean Difference (95% CI)	*P*-value
**Learning Curve – Communication score change (%)**	+ 14.2 ± 7.5	+ 6.5 ± 6.8	+ 7.7 (3.2–12.2)	0.012
**Learning Curve – Decision accuracy change (%)**	+ 12.8 ± 8.1	+ 4.9 ± 7.6	+ 7.9 (3.0–12.8)	0.021
**Reflection quality score (1–5)**	4.1 ± 0.6	3.5 ± 0.7	+ 0.62 (0.48–0.76)	< 0.001
**NASA-TLX Global workload**	47.2 ± 12.5	52.9 ± 13.1	–5.7 (–8.9 to − 2.5)	0.001
Frustration subscale	42.3 ± 11.8	50.4 ± 12.2	–8.1 (–11.5 to − 4.7)	< 0.001
Temporal demand subscale	46.0 ± 12.0	52.4 ± 12.5	–6.4 (–10.6 to − 2.2)	0.004

## Supplementary Information

Below is the link to the electronic supplementary material.

Supplementary Material 1

## Data Availability

The datasets generated and/or analyzed during the current study are available from the corresponding author upon reasonable request.
