# Supplementary material for: Effects of ChatGPT-generated immediate feedback integrated into VR-based OSCEs on nursing students’ performance: a randomized crossover study
Source: BMC Nurs. 2026 Apr 13;25:396. doi: 10.1186/s12912-026-04633-9 (PMC13112760; doi:10.1186/s12912-026-04633-9)
Supplement: Supplementary file 1 — Supplementary Material 1 [file 12912_2026_4633_MOESM1_ESM.docx]

**Supplementary Material S1. Validity and Reliability of Measurement Instruments**

**1. OSCE Communication Performance Scale**

Communication performance was assessed using a structured rating scale adapted from the Calgary–Cambridge Guide to medical communication. The Calgary–Cambridge framework is widely recognized for its strong content validity and construct validity in evaluating clinical communication skills across health professions education.
In the present study, two trained OSCE examiners independently rated each participant’s performance. Inter-rater reliability was evaluated using the intraclass correlation coefficient (ICC, two-way random effects, absolute agreement), which demonstrated excellent reliability (ICC = 0.89).

**2. Clinical Decision-Making Accuracy Checklist**

Clinical decision-making accuracy was measured using a scenario-specific checklist developed by a panel of three senior nurse educators based on established clinical guidelines. Content validity was ensured through expert consensus review.
Each checklist item was scored dichotomously (correct/incorrect), and overall decision accuracy was calculated as the percentage of correctly completed items. Given the criterion-based nature of the checklist, internal consistency indices were not applicable.

**3. Learning Satisfaction Scale**

Learning satisfaction was assessed using a 5-item Likert-type scale commonly employed in simulation-based education research. Previous studies have supported the construct validity of this instrument.
In the current sample, internal consistency reliability was good, with a Cronbach’s alpha coefficient of 0.87.

**4. AI Acceptance Questionnaire**

Acceptance of AI-assisted feedback was evaluated using an adapted Technology Acceptance Model (TAM) questionnaire, including perceived usefulness, perceived ease of use, and behavioral intention dimensions. The TAM has been extensively validated in educational technology research.
In this study, the overall scale demonstrated excellent internal consistency (Cronbach’s alpha = 0.91).

**5. Self-Efficacy Measures**

Academic self-efficacy was measured using the General Self-Efficacy Scale (GSES), which has been widely validated across diverse populations and cultural contexts. Communication self-efficacy was assessed using a scenario-specific scale developed for OSCE training contexts.
Internal consistency reliability in the present study was acceptable to high for both measures (Cronbach’s alpha = 0.85 for academic self-efficacy and 0.88 for communication self-efficacy).

**6. Reflection Quality Rating Rubric**

Reflection quality was evaluated using a structured 5-point rubric assessing descriptive clarity, analytical depth, and integrative reflection. Content validity was established through expert review by senior nursing educators.
Two independent raters scored all reflections, and inter-rater reliability was high (ICC = 0.82).

**7. Cognitive Workload (NASA Task Load Index)**

Cognitive workload was assessed using the NASA Task Load Index (NASA-TLX), a widely used and validated instrument in simulation, ergonomics, and human–computer interaction research.
In the present study, the NASA-TLX demonstrated satisfactory internal consistency (Cronbach’s alpha = 0.86).
